# Topical Inserts: A Versatile Delivery Form for HIV Prevention

**DOI:** 10.3390/pharmaceutics11080374

**Published:** 2019-08-01

**Authors:** M. Melissa Peet, Vivek Agrahari, Sharon M. Anderson, Homaira Hanif, Onkar N. Singh, Andrea R. Thurman, Gustavo F. Doncel, Meredith R. Clark

**Affiliations:** CONRAD, Department of Obstetrics and Gynecology, Eastern Virginia Medical School, Arlington, VA 22209, USA

**Keywords:** vaginal drug delivery, rectal drug delivery, HIV, tablets, protection, microbicides, fast-disintegrating, on-demand, extended-release

## Abstract

The development of topical inserts for the prevention of sexually transmitted infections (STIs), particularly human immunodeficiency virus (HIV), represents a promising alternative to oral and parenteral pre-exposure prophylaxis (PrEP) dosage forms. They may be used for vaginal and/or rectal administration of a variety of agents with antiviral activity. Topical inserts deliver drugs to the portal of viral entry, i.e., the genital or rectal mucosa, with low systemic exposure, and therefore are safer and have fewer side effects than systemic PrEP agents. They may dissolve fast, releasing the active drugs within minutes of insertion, or slowly for long-acting drug delivery. Furthermore, they are user-friendly being easy to administer, discreet and highly portable. They are also economical and easy to manufacture at scale and to distribute, with excellent stability and shelf-life. Altogether, topical inserts represent a particularly promising form of drug delivery for HIV and STI prevention. Highlighted within this review are end-user acceptability research dedicated to understanding preferred attributes for this form of drug delivery, advantages and disadvantages of the formulation platform options, considerations for their development, clinical assessment of select placebo prototypes, future directions, and the potential impact of this dosage form on the HIV prevention landscape.

## 1. Introduction

Globally, human immunodeficiency virus (HIV) remains a major public health crisis, with approximately 1.8 million new infections annually, and almost 38 million people living with HIV in 2018 [1]. New HIV infections are declining annually, but not rapidly enough to meet the 90-90-90 target (90% diagnosed, 90% on treatment, and 90% virally suppressed) by 2020, set by The Joint United Nations Programme on HIV and AIDS (UNAIDS) to end the acquired immune deficiency syndrome (AIDS) epidemic [2]. In order to meet current targets geared toward reducing new HIV infections to fewer than 500,000 globally by 2020, one of the highest priorities in the HIV prevention field is to develop stable, safe, effective and acceptable products which can successfully reduce HIV sexual transmission [3]. 

Oral pre-exposure prophylaxis (PrEP), specifically daily tenofovir disoproxil fumarate (TDF)/emtricitabine (FTC) (Truvada^®^), is highly effective when taken consistently [4,5,6]; however, adherence has proven challenging, especially in younger populations [7,8]. Recently, the 1-month dapivirine (DPV) intravaginal ring (IVR) was also proven to reduce HIV incidence. Protection was observed in women over 21 years, but not in younger women where adherence was lower [6,9]. The tenofovir (TFV) 1% gel, a 4-mL vaginal gel packaged in pre-filled applicators, demonstrated initial success in the CAPRISA 004 trial [10]; however, subsequent Phase IIb/III trials of the gel were unable to confirm effectiveness by intention-to-treat analysis in either daily [7] or pericoital (one dose before plus one dose after sex) [11] dosing regimens likely due to inconsistent and insufficient gel use in young, at-risk women. In an effort to minimize user dependency and thus potentially improve adherence, the development landscape in HIV prevention has recently pivoted to focusing on long-acting (LA) parenteral PrEP products, such as the cabotegravir injectable (CAB LA) currently in Phase III trials and several implant systems in preclinical development [12,13,14]. Although long acting parenterals have distinct benefits, they require health care provider delivery and training, have injection site and potential systemic side effects, and cannot be easily removed should safety concerns arise, all of which present potential new barriers affecting end-user uptake and continued use [15]. For users who desire an on-demand HIV prevention option that they can self-administer—easily, quickly, painlessly and discreetly—the development of user-friendly, topical dosage forms remains a global imperative. 

Delivering drugs via the vagina and rectum has several unique advantages, including increased local absorption of drug substances due to large surface area and rich blood supply and enhanced bioavailability, with potential for reduced systemic side effects [16,17]. When considering developing a product for the prevention of sexual transmission of HIV acquisition, delivering antiretrovirals (ARVs) topically provides a direct, first-line defense against HIV infection via genital/rectal mucosa. Therefore, topical dosage forms represent a promising delivery method. Dosage forms such as tablets/inserts, capsules, creams, suppositories, pessaries, foams, gels, films, rings, and douches have been pursued for vaginal and/or rectal delivery of drugs to improve sexual and reproductive health [18]. In this review, we will focus on topical (vaginal and rectal) inserts as a particularly promising and malleable delivery system for HIV prevention. Specifically, we will highlight the end-user acceptability research dedicated to understanding preferred attributes for this form of drug delivery, advantages and disadvantages of the formulation platform options, considerations for their development, clinical assessment of select placebo prototypes, and future directions and the potential impact of this dosage form on the HIV prevention landscape. 

## 2. Overview of Inserts for Female Reproductive Health 

### 2.1. Standardizing the Nomenclature 

Tablets are solid dosage forms primarily intended for oral administration. They are the most commonly used solid dosage form, as they are low cost, easy to manufacture and package, convenient for self-dosing, transportation and storage, and typically offer favorable stability and shelf-life profiles. “Inserts” are a type of tablet or other solid dosage form (e.g., ovule, soft gel capsule) that are intended for placement and retention in a body cavity, such as the vagina or buccal cavity. Per the USP Nomenclature Guidelines [19], suppositories are differentiated from vaginal inserts as being for rectal administration only, and are typically wax-based and melt, soften and/or dissolve at body temperature. However, as discussed later in this review, vaginal inserts under development specifically for HIV prevention may also be designed for rectal use, and therefore we have adopted the term “topical insert” to describe this “dual-compartment” class of drug delivery system.

Topical inserts, depending on the formulation technology used, may release drug either rapidly or slowly via one of several drug delivery mechanisms (e.g., insert disintegration, dissolution, controlled release) into the surrounding cervicovaginal fluid and tissue. Further, depending on the physicochemical properties and mechanism of action of the actives used, vaginal inserts may function by distributing drug lumenally, to local target cells and tissue, and/or via systemic circulation. Like oral tablets, topical inserts are in general economical, readily manufacturable, stable and user-friendly. Unlike vaginal gels, inserts are compact and do not require an applicator for administration, and therefore may be packaged, stored and used discreetly. Together, topical inserts are a versatile and convenient dosage form. A few examples of the many FDA approved vaginal insert products are listed in Table 1. In Africa, vaginal inserts are also a familiar, over-the-counter dosage form, with clotrimazole vaginal tablets for treatment of yeast/fungal infections being one common example.

### 2.2. Inserts in Development for HIV Prevention

**Figure 1:**
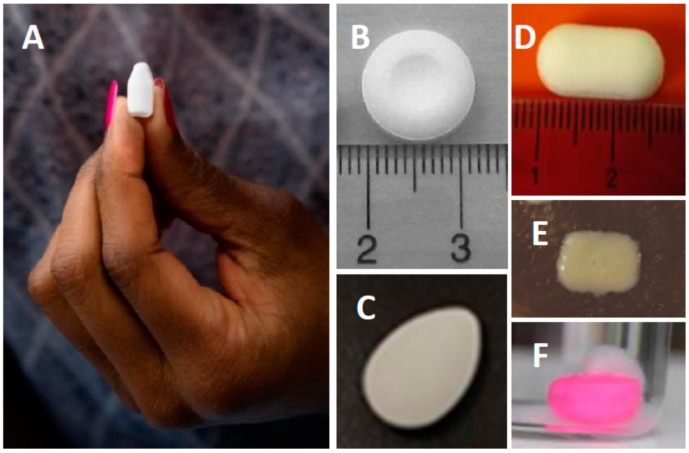
Representative images of topical inserts in development: (**A**) CONRAD’s Tenofovir Alafenamide Fumarate/Elvitegravir (TAF/EVG) insert, (**B**) CONRAD’s first generation insert, Tenofovir/Emtricitabine (TFV/FTC), (**C**) IPM’s DS003 vaginal tablet (photo courtesy of Jeremy Nuttall, IPM), (**D**) Population Council and PATH’s Griffithsin/Carrageenan (GRFT/CG) fast-dissolve insert (photo courtesy of Tom Zydowsky, The Population Council) [20], (**E**) Osel’s MucoCept *Lactobacillus* vaginal tablet (Reproduced from [21], under the terms of the CC BY 4.0 license), (**F**) prototype extended-release EVG osmotic insert, evaluated preclinically by CONRAD in collaboration with Patrick Kiser (Northwestern University, Evanston, IL, USA).

Although, topical inserts are used for a range of sexual and reproductive health indications, only a few research and development (R&D) groups are actively advancing topical inserts to clinical stages of development for HIV or multipurpose STI prevention (Table 2, Figure 1). However, topical inserts represent an excellent delivery platform for potent antiviral agents, especially when used as the basis for on-demand, event-driven pre- and post-exposure prophylaxis. With the incorporation of end-user research into the early product development process, inserts have great promise as a potentially safe, effective and highly desirable HIV prevention method option for a variety of target users. 

## 3. Insert Platforms, Technologies and Mechanisms of Dissolution

Depending on the type of formulation technology used and mechanism of action of the specific drug(s) to be delivered, topical inserts may be designed to quickly disintegrate or dissolve within the vagina or rectum to release therapeutic drugs quickly for on-demand (event-driven) use, or provide an extended/controlled drug release through bioadhesive properties that may prolong retention and pharmacokinetics (PK). 

**Figure 2:**
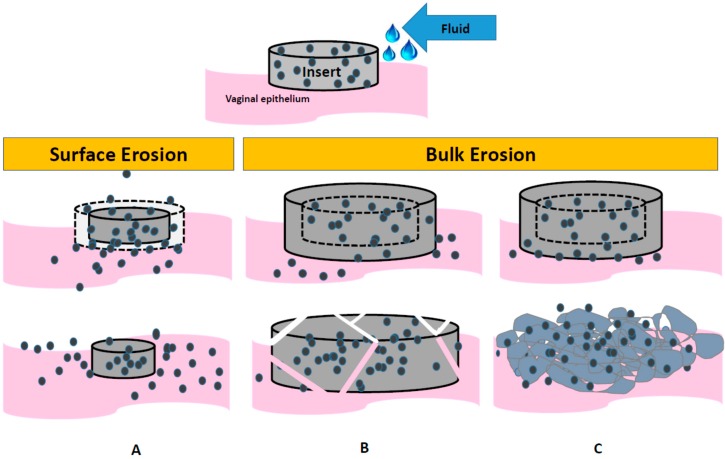
Schematic of the different processes of topical insert disintegration with the introduction of vaginal fluid. Surface erosion: (**A**) The size of the insert decreases as the surface disintegrates releasing drug. Bulk erosion: The size of the insert first increases due to fluid uptake and swelling, followed by (**B**) breakdown of the insert into smaller pieces until fully disintegrated or (**C**) transformation from the solid state into a gel.

The mechanism and rate of insert disintegration is a complex phenomenon, dependent upon the excipient and drug properties and vaginal fluid, which varies further in pH, composition and volume depending on a woman’s age, menstrual cycle and microbiota. Disintegration may follow a process of bulk or surface erosion, or a combination of both. Also depending on the excipients used, an insert may dissolve or disintegrate into the vaginal fluids or transform into a gel. In general, fast-disintegration requires rapid absorption of water into the core of the insert creating pores for faster dissolution of the inserts. In the case of surface erosion, an insert erodes from the surface creating a loss in mass that is faster than the absorption of water into the bulk of the product (Figure 2A). With bulk erosion, the absorption of water is faster than the rate of disintegration, often swelling the insert before breaking the insert into increasingly smaller (higher surface area) units that facilitate drug release and further disintegration (Figure 2B), or alternatively transforming the insert into a hydrated gel form (Figure 2C).

In the following sections, we describe the different types of insert technologies, products and platforms in development and/or consideration for HIV prevention.

## 4. Fast Dissolving Inserts 

Fast in vivo degradation and drug release are critical for the development of a rapidly-disintegrating topical microbicide insert platform. Common classes of excipients used to make fast-dissolving inserts include: Disintegrants (superdisintegrants), diluents, binding agents, dispersing agents, glidants, osmoattractants, buffering agents, lubricating and pore formers or channel creators. The major technologies for manufacturing fast-dissolving/disintegrating inserts are direct compression and freeze-drying which have been primarily used for vaginal/rectal insert manufacturing and discussed here.

### 4.1. Direct Compression Inserts

Direct compression is a process involving drug-excipient blending, typically by either direct blend or dry/wet granulation methodologies, followed by compaction using a tablet press. Compared to other methods of insert manufacturing, direct compression has several advantages. It is a simple, robust and cost-effective method with a limited number of processing steps involved. It allows for the production of a less-friable insert with high mechanical strength, thus better handling and packaging feasibility. The disadvantages of this process include the manufacturing of less porous inserts, which means a longer dissolution time is likely needed for dissolution or disintegration, especially when compared to freeze-dried inserts. Dissolution rate is affected by the level of compression, which affects the porosity and mechanical strength or hardness of the insert. Therefore, an appropriate balance is needed for achieving sufficient porosity (for fast disintegration/dissolution) and mechanical strength (to adequately prevent premature breakage during manufacture, packaging and storage).

CONRAD’s topical insert development program for HIV/STI prevention has focused primarily on the use of the compressed insert platform, due to ease of manufacture, low cost, and compatibility with different ARVs. Our first generation product (Figure 1B) was intended as “topical Truvada” and combined the nucleos(t)ide reverse transcriptase inhibitors TFV and FTC [22,23]. This first-generation product was tested in a Phase I trial, CONRAD 117 (NCT01694407), and while safety and PK benchmarks were met, the product was slow to disintegrate and resulted in a leakage of white, insoluble residual matter [24]. CONRAD’s development has since advanced, with significant end-user feedback (see below), to a next-generation, dual-compartment product (Figure 1A) combining the TFV prodrug tenofovir alafenamide fumarate (TAF) with elvitegravir (EVG), an advanced integrase strand-transfer inhibitor, this time in a surface-erosion type formulation that dissolves with minimal residue. The TAF/EVG combination provides a more potent combination with a wide pre- and post-coital window of prophylaxis, against both HIV and herpes simplex virus (HSV), supportive of flexible, pharmacologically forgiving, on-demand PrEP or postexposure prophylaxis (PEP) dosing. Accelerated and long-term stability testing at 40 °C/75% RH and 30 °C/65% RH demonstrate the product to be physicochemically stable, and dose-ranging rabbit vaginal and rectal irritation studies support its safety for clinical use. Preclinical proof-of-concept for preventing vaginal transmission of SHIV in non-human primates has been demonstrated for this product [25]. Interestingly, the TAF/EVG combination product was able to prevent infection even when administered after viral exposure. A first-in-women study (CONRAD 146, NCT03762772) evaluating the safety, PK and pharmacodynamics (PD), for both HIV and HSV, of the vaginally administered TAF/EVG insert was recently completed with data expected in late 2019. MTN 039, a safety/PK study to evaluate the TAF/EVG insert following rectal dosing, is expected to initiate in late 2019 [26].

The International Partnership for Microbicides (IPM) is developing a compressed, fast-dispersing tablet for the vaginal delivery of DS003 (Figure 1C), a highly hydrophobic gp120 blocker, alone and in combination with dapivirine [27,28]. While IPM’s primary purpose of developing the insert form was to expedite getting DS003, a new chemical entity, into first-in-human testing to support further development in a sustained-release vaginal ring form, the luminally active entry inhibitor mechanism of DS003 provides strong rationale for the DS003 vaginal tablet as a promising on-demand product. The DS003-only vaginal tablet was evaluated clinically in IPM 042 (NCT02877979), a dose-escalating Phase I study that completed in 2016 and reported the product to be safe, well tolerated and capable of achieving PK levels in cervicovaginal fluids sufficient to provide antiviral activity ex vivo [27,28].

### 4.2. Freeze-Dried Inserts

Compared to compressed inserts, freeze-dried inserts are highly porous structures that allow for rapid dissolution or disintegration of the dosage form even in very low fluid volume environments, such as the vagina. The process to make freeze-dried inserts involves dissolving or dispersing the actives and excipients in aqueous solutions, pouring this solution into an open mold (typically a blister pack type mold), then lyophilizing to form the final solid dosage form. In addition to being very fast dissolving, the freeze-dried manufacturing process uses low temperatures, minimizing adverse thermal effects that may affect drug stability, and results in amorphous structures of excipients and drug, leading to enhanced dissolution. For biologics especially, this method of manufacturing may improve product stability [29,30]. However, some potential limitations of the freeze-dried insert platform include: (a) It is a relatively expensive manufacturing process due to increased complexity of the equipment; (b) formulations may require addition of a cryoprotectant to maintain drug stability during processing; (c) hygroscopicity of the product can lead to poor stability at higher temperatures and humidity, requiring special care for packaging, storage, and handling of the product; (d) weak mechanical properties may make the product too fragile to withstand handling during removal from packaging and vaginal insertion; e) limited drug loading capacity; and (f) drugs must be chemically stable [29,30].

During CONRAD’s early development of inserts for vaginal delivery of ARVs, we developed a freeze-dried, rapidly dissolving insert using Catalent’s Zydis^®^ fast-dissolving technology platform original developed for oral drug delivery [31]. Prototype inserts containing TFV, TDF and/or EVG were investigated in vitro. Clinical assessments of a placebo freeze-dried insert (in CONRAD 134, NCT02534779) determined the product easy to insert vaginally and completely dissolved in the vaginal lumen within 30 min, providing the first clinical evidence that a freeze-dried insert formulation may be robust enough for vaginal administration (see Section 7.2 below for additional discussion on CONRAD 134) [31,32]. While these results were promising, due to primarily scale up cost considerations, CONRAD selected the compressed insert platform for continued advancement of the TAF/EVG insert.

The most advanced freeze-dried vaginal insert in development for prevention of HIV and sexually transmitted infections (STI) is the Griffithsin (GRFT)/Carrageenan (CG) fast-dissolve insert from Population Council in collaboration with PATH [20,33]. GRFT is a naturally-occurring algae-derived protein that inhibits HIV and other pathogens, including herpes simplex virus (HSV-2); CG is a sulfated polysaccharide with potency against human papilloma virus (HPV). The GRFT/CG fast-dissolve inserts disintegrated in <1 min in a physiologically relevant volume (∼1 mL) of vaginal fluid, transforming rapidly into a viscous, spreading gel with mucoadhesive properties [34]. In macaques, the GRFT/CG inserts demonstrated protection in a high dose vaginal SHIV challenge study 4 h after insert insertion, and in mice, the inserts protected against vaginal HSV-2 and HPV pseudo virus [33,35]. While clinical testing of the GRFT/CG insert has yet to be conducted, a Phase I study (NCT02875119) of a GRFT/CG gel formulation was completed in 2018, providing first-in-human safety, PK and PD data for GRFT dosed vaginally.

Osel, a developer of live biotherapeutic products, reported preclinical development findings on a vaginally disintegrating freeze-dried insert formulation containing genetically engineered *Lactobacillus* jensenii that secrete a modified form of the potent HIV-1 entry inhibitor protein, Cyanovirin-N (the MucoCept^®^ technology platform, Figure 1E) [21]. These inserts disintegrate within 2 min in vitro and are reported as stable at 4 and 25 °C. Vaginal administration of inserts to rhesus macaques resulted in colonization of the MucoCept *Lactobacillus* in a majority of macaques after 14–21 days and effectively released measurable cyanovirin-N into vaginal fluids [21]. These results are encouraging, as they demonstrate preclinical proof-of-concept that a convenient solid, freeze-dried insert dosage form may be a feasible and stable approach to delivering biotherapeutic agents topically.

## 5. Extended-Release Inserts

While the most advanced inserts in development for HIV prevention are intended to be fast dissolving/disintegrating, extended-release inserts may be an approach to leverage this simple, user-friendly insert dosage form to provide less coitally-dependent dosing and sustained PK topically (and/or systemically) for a longer, “intermediate” duration (e.g., several days to few weeks) compared to, say, intravaginal rings that last one or more months. A large question mark for the clinical feasibility of extended-release inserts, however, is whether vaginal retention of these products will be sufficiently long, especially during ambulation, physical activity and coitus. Evidence from studies with intravaginal rings and vaginal films suggest presence of the vaginal product during sex is acceptable [36,37,38,39,40], however such studies with an extended-release insert will be necessary. Below we summarize some of the main formulation approaches investigated for this insert class. 

### 5.1. Mucoadhesive Matrix-Based

The simplest of the extended-release formulation approaches is through the incorporation of mucoadhesive polymers in either compressed or hot-melt extruded inserts. In a collaboration with Dr. Karl Malcolm (Queen’s University Belfast, Belfast, UK) and CONRAD, a sustained-release hydroxypropyl methylcellulose (HPMC)-based mucoadhesive TFV vaginal insert formulation was developed using a direct compression technique [41]. By varying the type and molecular weight of HPMC, in addition to other variables, the drug release rate was modulated in vitro. More recently, work led by María Dolores Veiga (Universidad Complutense de Madrid, Madrid, Spain) describe the in vitro and ex vivo formulation development of several sustained ARV-releasing, mucoadhesive insert formulations for HIV prevention, including for TFV, TDF and dapivirine, using various direct compression, granulation, and hot-melt extrusion techniques and polymer excipients [42,43,44,45,46,47]. 

### 5.2. Osmotic-Controlled

Osmotic pump-like inserts are polymer-coated inserts containing a semi-permeable, non-swellable outer membrane with a small, typically laser-drilled, orifice. These systems are formulated with osmoattractants in the tablet core to drive water to diffuse through the outer membrane. In turn, the osmotic pressure increases in the core, which is constrained by the rigid membrane, and drives the sustained release of drug out through the drug delivery orifice. One of the advantages of these systems is that the release mechanism is largely drug-independent, with a high degree of in vitro and in vivo correlation. However, the more complex manufacturing and thus higher cost than conventional tablet technologies, as well as potentially a higher risk of dose dumping if the coating were to rupture or otherwise fail, represent known limitations of osmotically controlled technologies. Dr. Patrick Kiser (Northwestern University) first reported on the application of osmotic pump technology in a sustained-release microbicide vaginal insert containing the pyrimidinedione analog IQP-0528 [48]. Following a single vaginal dose in sheep, IQP-0528 osmotic pump inserts were demonstrated to achieve consistent drug levels for up to 10 days in the vaginal fluid and mucosa, whereas uncoated inserts depleted their payload within 48 h, suggesting that the osmotic pump topical insert platform has the potential to achieve long-acting drug release profiles for HIV prevention. In a collaboration between Dr. Kiser and CONRAD, this platform was further investigated for sustained EVG delivery (Figure 1F); a pilot study conducted in rhesus macaques (with Ron Veazey (Tulane University)) suggested prolonged retention and sustained vaginal PK of EVG for at least one week is feasible from this system, however further development would be needed to optimize disintegration of the drug-spent device and evaluate the impact of coitus on vaginal retention and PK. 

### 5.3. Integration of Multiparticulate Drug Delivery Systems 

Multiparticulate-based drug delivery systems have the potential to achieve several properties that may be desirable for topical PrEP insert formulations, including potential for drug targeting to specific cells or mucosal tissue, increased mucous and tissue penetration, bioadhesion, and retention, and ability for sustained, controlled and/or stimuli-triggered drug release after vaginal administration [49,50,51,52]. As has been investigated for other applications, drug-encapsulated nano/microparticles can be compressed into pellets or inserts as the final dosage form [53,54]. To date, incorporation of sustained-release multiparticulate drug delivery systems into vaginal HIV prevention formulations has been limited to liquid suspension [51], gel [55,56,57,58] and film [59,60,61] dosage forms, and have yet to be incorporated into solid vaginal inserts. 

## 6. End-User Acceptability of Inserts for HIV Prevention

As mentioned earlier, product developers are increasingly including user feedback in early stages of development to help ensure that products are aligned with user preferences. Most clinical trials measure acceptability in some form, primarily via willingness to use in the future. In general, such studies have shown positive acceptability of vaginal microbicides. More recently, studies have examined preferences across products and focusing on specific attributes. These studies have used a mixed methods approach, including discrete choice experiments (DCE) and conjoint analysis, to understand preferences by comparing and weighing different attributes. Pre- and post- product use questionnaires can also provide qualitative and quantitative feedback on user preferences and rankings when comparing different dosage forms. Together, these studies have shown the variability of preferences for HIV prevention products across groups and geographies indicating the need for multiple delivery systems to address women’s needs and preferences [62,63]. 

In 2009, a consumer product preference study was conducted by IPM comparing a placebo vaginal film, soft-gel capsule and vaginal insert among women in Burkina Faso, Tanzania and Zambia (*n* = 526) [64]. While this study found all three forms to be acceptable, less than half of the women complied with precoital use (especially limited use in Burkina Faso). The preferred dosage form varied, with soft-gel capsule selected in Burkina Faso and Tanzania and film in Zambia. While most women found the vaginal insert’s size and color (white to off-white) as acceptable, the vaginal insert, which was a placebo form similar to IPM’s DS003 vaginal insert tested clinically, was reported to have several drawbacks including slow disintegration, rough texture, and leakage issues. However, most women stated they would use each product if it were efficacious, despite product leakage.

The recent Quatro study, a collaboration between CONRAD and RTI International, assessed preferences across four vaginal placebo products (film, insert, gel and ring) among women in Zimbabwe and South Africa using a randomized crossover design [63]. Products were ranked by participants at enrollment and after use. While at baseline the gel was selected as the most preferred product (41%) and the ring as least preferred (61%), rankings changed after use with no clear overall preference for one product (film 29%; ring 28%, insert 26%, and gel 16%). In South Africa, however, after the use phase, the CONRAD insert was chosen as the preferred product (35%). A DCE was also conducted as part of Quatro among product naïve and experienced participants [62]. While efficacy was the most important attribute, products that provided dual protection from pregnancy and HIV and some vaginal wetness were also preferred. For mode of insertion, a reusable applicator was preferred over a disposable applicator or insertion by finger.

A study with 68 former VOICE trial participants from South Africa, Zimbabwe and Uganda explored preferences for eight different potential microbicide dosage forms (oral inserts, vaginal gel, injectable, implant, vaginal ring, vaginal film, vaginal insert, cervical barrier/diaphragm) [65]. While the majority of participants preferred long acting methods, interest in on-demand methods was more likely among women from South Africa, women who did not live with a primary sex partner, those of lower parity and those completing secondary school or higher with higher socioeconomic status. Women preferred long acting or on-demand methods due to concerns about adhering to a daily regimen. Some concerns on ease of use were connected to familiarity, which dissipated with product experience. This is important to note given their choice was based on hypothetical products they may not have used. 

Currently there is an online survey from Population Council on women’s preferences for multipurpose prevention technologies (MPTs) and HIV/STI prevention methods to understand preferred attributes but also to see if women can be grouped by needs and preferences. While the survey is not complete, it has reached more than 700 women across 50 countries. As of May 2019, a fast dissolving insert (64%) was the most selected method, with gel before sex (63%) and pill before sex (62%) close behind [20]. 

Building on this growing evidence from end-user research that vaginal inserts are a promising and acceptable dosage form for HIV prevention, studies have also been conducted to further optimize the desirability of inserts. Throughout the development of the CONRAD vaginal insert, for example, acceptability data from clinical use and human-centered design studies were used to inform iterative development of the lead formulation and form. Overall, the insert has been found easy to use and highly acceptable, especially after reformulation between first and second generation products to minimize discharge or leakage (see Section 7.2 below). The form of the insert has also changed over time based on user input from both qualitative data from the CONRAD 117 study (some users likened the 1st generation insert to an oral breath mint) and a human centered design study conducted in South Africa. Based on the learnings and design principles from this latter research (manuscript pending), the shape and size of the insert were modified to provide directionality and thereby make it more intuitive for vaginal use and differentiated from oral pills and tablets, as well as easier to hold and insert by hand. Additionally, single-dose packaging concepts have been developed for the vaginal insert, to enable improved portability, discreet use, and desirability among particularly South African young women.

## 7. Considerations for the Development of Inserts for HIV Prevention

**Figure 3:**
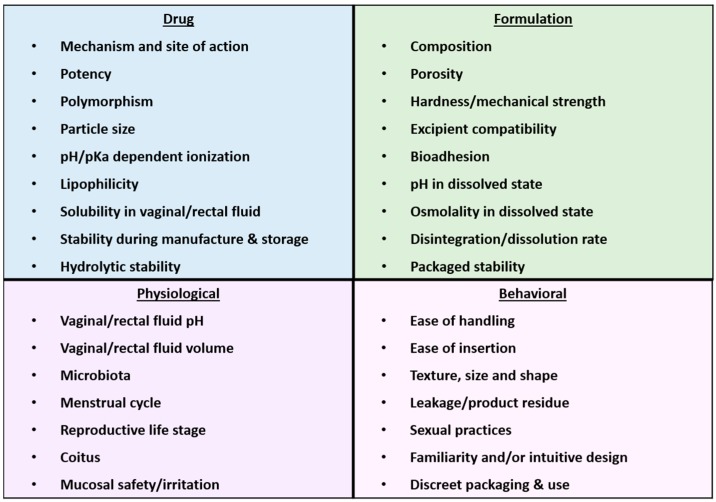
Factors to consider during the design and development process of inserts.

As alluded to early in this review, there are a number of factors to take into account when designing and developing an insert for HIV prevention. Key points under each of these factors are presented below in Figure 3.

Potency, duration and mechanism of action of the drug(s) to be delivered are important. For example, Population Council’s Griffithsin is a lumenally-active entry inhibitor that must be delivered rapidly to the vagina and retained through the period of potential HIV exposure (coitus); a fast-dissolve, freeze-dried insert that dissolves into a gel form is therefore highly appropriate for this target product profile. For CONRAD’s TAF/EVG insert, however, these two drugs require tissue uptake and, in the case of TAF, intracellular metabolism to the active TFV-diphosphate. For these drugs, a simple compressed insert formulation may be sufficient to enable rapid drug permeation into the surrounding tissues. Understanding the target population and sexual practices of that population is equally important. If a product can be used vaginally as well as rectally, such as the design of CONRAD’s TAF/EVG insert, this may widen the prophylactic coverage across multiple at-risk populations, providing further value added. Physiological factors, including changes or differences in vaginal pH, menstrual cycle, hormone levels, vaginal fluid volume, and stage in the reproductive cycle (pre, peri, or menopausal), must be considered for their effects on insert dissolution/disintegration and drug distribution and absorption [66]. Moreover, the insert should not cause changes in the vaginal microbiota or irritate the mucosal tissue. Due to the mechanisms of how inserts dissolve or disintegrate from a solid dosage form to a semisolid or liquid form—these factors may impact the insert’s performance. Below we summarize the types of models and study designs used by CONRAD and others to evaluate ARV insert formulations and performance in vivo.

### 7.1. Animal Models 

Arguably the best animal model for evaluating the safety, PK, PD and efficacy of a candidate vaginal insert formulation for HIV prevention is the non-human primate (NHP) model. Of the macaque species used in HIV prevention R&D [67], rhesus and pigtailed macaques have primarily been used to evaluate the PK and prophylactic efficacy of topically administered anti-HIV microbicides including inserts. Pigtail macaques are typically used for vaginal drug application and the vaginal transmission of virus [23,68,69] due to their lunar menstrual cycling; however, rhesus macaques treated with depot medroxyprogesterone acetate (DMPA) to thin the vaginal epithelium are an alternative and often more accessible species [70]. Due to the size of these macaques, topical microbicides including inserts may be used in their clinical and/or miniaturized form allowing for a more direct correlation between animal and human studies. Systemic exposure in plasma and local exposure in mucosal fluid and tissues may be collected to assess multi-compartmental PK profiles. Prophylactic efficacy may be assessed using various vaginal or rectal dosing regimens in NHPs, including PrEP or PEP treatment regimens, and single or repeated dosing and challenges with simian or simian-human immunodeficiency virus (SIV or SHIV) strains. In addition, using macaques or other large animals for evaluation of fast-dissolve inserts provides the opportunity to monitor insert disintegration in vivo via colposcopy. 

Small animals are also commonly used to evaluate inserts [22,35]. Disposition of drug systemically and locally can be evaluated in the rabbit using either intact or predissolved inserts. Due to their lack of infection with HIV [71], rabbits are not used in efficacy testing but are essential for assessing mucosal safety, toxicity and tolerability for the Investigational New Drug (IND)-enabling development of vaginal microbicides. Eckstein et al. developed a vaginal irritation scoring model and demonstrated that the rabbit was better suited for vaginal tolerance tests of spermicidal preparations than macaques as results in the rabbit were considered more sensitive and more consistent with clinical data than that of the monkey [72]. The rabbit has since been the preferred species for vaginal, as well as rectal irritation tests [73]. Humanized mouse models may also be an option for assessing both PK and HIV prophylaxis [74], however highly miniaturized inserts are required for vaginal dosing.

CONRAD has used both macaque and rabbit models in their effort to advance and guide the development of ARV containing inserts and define their safety and PK profiles. Rabbit and pigtail macaque models were used to assess the local and systemic PK of the first-generation TFV/FTC vaginal inserts compared to 1% TFV gel, with similar PK patterns observed across species [22,23]. The TAF/EVG inserts currently in clinical development have been assessed for safety, PK, PD and prophylactic potential against vaginal SHIV challenges in pigtailed macaques through a collaboration with the Centers for Disease Control and Prevention (CDC) [25]. In addition, we routinely conduct vaginal irritation and local tolerability testing in good laboratory practice (GLP) rabbit studies prior to Phase I clinical testing, as well as non-GLP assessments of insert disintegration, vaginal pH, local inflammatory cytokines, and microflora as part of the pigtailed macaque studies. Together, these studies are used to support the nonclinical portion of the IND submission to the FDA. 

Population Council has used a similar approach to demonstrate the safety and efficacy of their non-antiretroviral product, GRFT/CG insert. The rabbit model was used to evaluate local vaginal irritation. The rat model was used to evaluate vaginal irritation and test for systemic absorption and anti-drug antibodies [35]. The GRFT/CG insert has also been tested in a high dose vaginal SHIV challenge model in rhesus macaques treated with DMPA, as well as mouse HSV-2 and HPV challenge models, to demonstrate the on-demand MPT properties of this insert [35].

Animal models can pose limitations, however, for accurately predicting the disintegration and dissolution time of inserts clinically, as discovered during CONRAD’s development of the first-generation TFV/FTC insert product. When tested in both the rabbit and macaque, the TFV/FTC vaginal insert was no longer present at 30 min; however, when tested clinically in women, CONRAD 117, the insert was still intact at 30 min in over 50% of the 48 participants [24]. For at least this compressed insert formulation, which contained superdisintegrants to enable “rapid disintegration”, the rabbit and macaque were not a good predictor of what was observed clinically for that particular formulation. These differences across species may have been specific to one or more variables such as excipient and drug properties or manufacturing process of the insert. Our effort to develop a standardized, reliable method to predict dissolution or disintegration of inserts in vitro have also been challenged due likely to the complexity of how different compositions and insert technologies are designed to dissolve or disintegrate (as described above in Figure 2). It is therefore important to take these factors into consideration when defining the best preclinical models for evaluating the performance of a given insert formulation.

### 7.2. Incorporating Clinical Studies of Placebo Formulations into Early Product Development 

**Figure 4:**
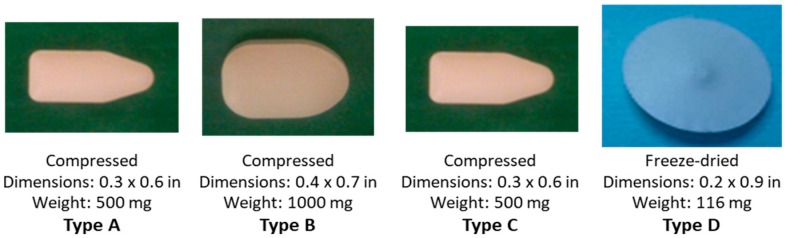
Placebo vaginal inserts tested in CONRAD Study D15-134 [32].

In CONRAD’s experience with compressed inserts, nothing has proven more effective in the confirmatory development of a new formulation than early clinical assessment of placebo formulations. As previously discussed, CONRAD’s first-generation vaginal inserts failed to replicate what had been observed in vitro and in animal models when tested clinically. Therefore, when evaluating the disintegration, residue leakage and acceptability profiles of reformulated inserts, we turned to a small clinical study to inform our lead selection decision making. In CONRAD 134 (NCT02534779) a clinical study conducted at the CONRAD/Eastern Virginia Medical School (EVMS) Clinical Research Center, four placebo vaginal inserts of different composition, technology (compressed v. freeze-dried), shape and/or size (Figure 4) were investigated in 32 women (age 18–45 years), with the primary objective of assessing the time needed for disintegration and complete disappearance, and secondarily, the acceptability after a single use. Vaginal inserts were assessed first during an in-clinic disintegration assessment, and a second time as part of an at-home acceptability assessment. 

In this study, all four of the improved insert prototypes studied in CONRAD 134 disintegrated faster and had enhanced acceptability over our first-generation inserts in CONRAD 117. Inserts showed rapid initial disintegration, occurring in most cases within 15 min after insertion, with the freeze-dried insert (Type D) being the fastest to completely disappear, doing so within 30 minutes after insertion in all women. Across all groups, minimal to no leakage or product residue was reported. Product acceptability, as assessed by a standardized acceptability questionnaire, focused on two key attributes of the insert: (1) Ease of handling and placement of the vaginal insert, and (2) leakage. Overall, the vaginal insert types were found to be highly acceptable, with a majority of participants reporting they “definitely would” or “probably would” use the insert if it were shown to be effective in preventing HIV and they felt they were at risk. Insertion was rated “very easy” or “fairly/somewhat easy” by 100% of participants in this study. Most of the women (83.3–91.7%) reported that the more directional shape (Types A, B, or C) versus the biconcave disk (Type D) was helpful for insertion. About three-quarters of the users of each insert type felt the size was appropriate, with a greater proportion favoring the Type A and C size over the larger Type B and indicated they did not feel that a smaller insert would be easier to insert. Together, the results from CONRAD 134 provided clinical evidence supporting the continued development of a formulation and form similar to Type C [32], for the TAF/EVG insert now in clinical development.

## 8. Future Directions and Potential Challenges 

Topical inserts are a promising, user-friendly dosage form for vaginal and/or rectal, on-demand prophylaxis against HIV and/or other STIs. Several product candidates, each employing unique antiviral modalities and drug combinations, are currently in late preclinical or early clinical development, with increasing evidence supporting the acceptability of this highly versatile dosage form. Through incorporation of extensive user feedback, the insert formulations advancing today align with stated user preferences, including how they may better fit into users’ lifestyles and therefore provide potential to more effectively expand the HIV prevention method mix with a discreet, portable, low cost and potentially forgiving on-demand or extended, intermediate dosing regimen. Some of these products are also uniquely suited for developing country manufacturing, storage and distribution. With preclinical proof-of-concept demonstrated for the leading insert candidates in development, and Phase I testing recently completed for some, the greatest challenge for this dosage form is securing the continued support and R&D prioritization amongst potential funding stakeholders to enable further development and clinical advancement of the most promising HIV prevention insert products.

Although the current trend in HIV prevention R&D is to develop long-acting systemic formulations, topically applied inserts represent an on-demand option for HIV-negative men and women who do not like to be constantly exposed to HIV drugs, experience side effects, or prefer user-controlled methods. Topical inserts would also be a preferred option for individuals having infrequent sex or short patterns (“seasons”) of potential exposure to HIV and risk. 

Due to their malleability and the ability to deliver multiple drugs with different physicochemical properties, topical inserts are especially suited for combination methods and multi-indication products or MPTs. With STIs on the rise again, the ability to deliver multiple drugs at high concentrations in the compartments where the infections develop, i.e. cervicovaginal and rectal tracts, will make this dosage form a critical addition to the existing and in-development HIV/STI prevention tools.

## Figures and Tables

**Table 1:** Examples of FDA approved topical inserts.

Example Products	Indication
Mycelex-G	Treat vulvovaginal yeast (*Candida*) infections
Semicid, Encare^®^	Prevent pregnancy
Intrarosa^®^, Imvexxy^®^	Treat moderate-to-severe dyspareunia due to menopause
Endometrin^®^	Support embryo implantation in early pregnancy
Vagifem^®^	Treat vaginal irritation and dryness caused by menopause

**Table 2:** Topical inserts in development for HIV or multipurpose HIV prevention.

Product Name	Developer	Insert Technology	Indication	Development Phase
Tenofovir Alafenamide Fumarate/Elvitegravir Topical Insert	CONRAD/EVMS (Arlington and Norfolk, VA, USA)	Compressed	HIV, HSV (vaginal or rectal use)	Phase I
Griffithsin/Carrageenan Fast Dissolve Insert (PC-9500)	Population Council (New York, NY, USA) in collaboration with PATH (Seattle, WA, USA)	Freeze-Dried	HIV, HPV, HSV (vaginal use)	Preclinical *
DS003 Vaginal Tablet	IPM (Silver Spring, MD, USA)	Compressed	HIV (vaginal use)	Phase I
MucoCept^®^ *Lactobacillus* Vaginal Tablet	Osel (Mountain View, CA, USA)	Freeze-Dried	HIV (vaginal use)	Preclinical

* A gel form of Griffithsin/Carrageenan (GRFT/CG) combination is in Phase I clinical development stage. HSV, herpes simplex virus. HPV, human papilloma virus.

## Abbreviations

The following abbreviations are used in this manuscript:

AIDS	Acquired Immune Deficiency Syndrome
ARV	Antiretroviral
CAB	Cabotegravir
CDC	Centers for Disease Control and Prevention
CG	Carrageenan
DCE	Discrete Choice Experiment
DMPA	Medroxyprogesterone Acetate
DPV	Dapivirine
EVG	Elvitegravir
EVMS	Eastern Virginia Medical School
FTC	Emtricitabine
GLP	Good Laboratory Practice
GRFT	Griffithsin
HIV	Human Immunodeficiency Virus
HPMC	Hydroxypropyl Methylcellulose
HPV	Human Papilloma Virus
HSV	Herpes Simplex Virus
IND	Investigational New Drug
IPM	International Partnership for Microbicides
IVR	Intravaginal Ring
LA	Long-acting
MPT	Multipurpose Prevention Technologies
NHP	Non-human Primate
PD	Pharmacodynamics
PEP	Post-exposure Prophylaxis
PK	Pharmacokinetics
PrEP	Pre-exposure Prophylaxis
R&D	Research and Development
RH	Relative Humidity
SHIV	Simian-human Immunodeficiency Virus
SIV	Simian Immunodeficiency Virus
STI	Sexually Transmitted Infection
TAF	Tenofovir Alafenamide Fumarate
TDF	Tenofovir Disoproxil Fumarate
TFV	Tenofovir
UNAIDS	The Joint United Nations Programme on HIV and AIDS
